# High infiltration of CD68+ macrophages is associated with poor prognoses of head and neck squamous cell carcinoma patients and is influenced by human papillomavirus

**DOI:** 10.18632/oncotarget.24306

**Published:** 2018-01-24

**Authors:** Imelda Seminerio, Nadège Kindt, Géraldine Descamps, Justine Bellier, Jérôme R. Lechien, Quentin Mat, Charles Pottier, Fabrice Journé, Sven Saussez

**Affiliations:** ^1^ Department of Human Anatomy and Experimental Oncology, Research Institute for Health Sciences and Technology, Faculty of Medicine and Pharmacy, University of Mons, Mons B-7000, Belgium; ^2^ Department of Pathology, C.H.U., SART TILMAN, University of Liège, Liège 4000, Belgium; ^3^ Laboratory of Oncology and Experimental Surgery, Jules Bordet Institute, Université Libre de Bruxelles, Brussels 1000, Belgium; ^4^ Department of Oto-Rhino-Laryngology, Faculty of Medicine, Université Libre de Bruxelles (ULB), Brussels B-1000, Belgium; ^5^ Present address: Metastasis Research Laboratory, GIGA-Cancer, University of Liege, Liège 4000, Belgium

**Keywords:** head and neck squamous cell carcinoma, human papillomavirus, CD68, macrophages, mouse

## Abstract

Incidence of human papillomavirus (HPV)-related head and neck squamous cell carcinomas (HNSCCs) has increased over the last few decades. The reaction of the host immune system to these tumors remains biologically complex. Here, we investigated CD68+ macrophage numbers, reporting the prognostic value in comparison to other risk factors. We also examined CD68+ macrophage infiltration during disease progression regarding the impact of HPV infection, and we studied the role of HPV16-E6/E7 oncoproteins in CD68+ macrophage recruitment. CD68+ macrophage numbers were evaluated in 10 cases of tumor-free peri-tumoral epithelia, 43 cases of low-grade dysplasia, 45 cases of high-grade dysplasia and 110 cases of carcinoma. Our *in vivo* model was developed in 80 C3H/HeN mice orthotopically injected with HPV16-E6, -E7 or -E6/E7-transfected SCC-VII cell lines. High CD68+ macrophage numbers in the intra-tumoral compartment were associated with shorter patient survival (recurrence-free survival: *p* = 0.001; overall survival: *p* = 0.01). Multivariate analyses reported that CD68+ macrophage infiltration and tumor stage were strong and independent prognostic factors of HNSCC. CD68+ macrophage numbers increased during HNSCC progression both in intra-epithelial (*p* < 0.001) and stromal compartments (*p* < 0.001). A higher density of CD68+ macrophages was observed in advanced stages (*p* = 0.004). Patients with transcriptionally active HPV infections had higher CD68+ macrophage density than did HPV-negative patients (*p* = 0.003). CD68+ macrophage infiltration was higher in HPV-E7+ and −E6/E7+ mouse tumors than in -E6+ tumors (*p* = 0.029 and *p* < 0.001). In conclusion, the extent of CD68+ macrophage infiltration is a significant prognostic factor for HNSCC patients. The recruitment of macrophages increases during disease progression and is influenced by the HPV virus.

## INTRODUCTION

Head and neck squamous cell carcinoma (HNSCC) remains one of the most common cancers worldwide, with more than 550,000 new cases diagnosed each year [1, 2]. Tobacco and alcohol abuse is the main risk factor for these cancers, but infection with oncogenic human papillomavirus (HPV) also appears to be involved in head and neck carcinogenesis [3, 4]. Indeed, we observed an increasing proportion of HPV-infected HNSCCs in non-smoking and non-drinking young people (<45 years old) [5, 6]. However, there is a discrepancy between this subgroup of patients *versus* older (>45 years old) smoking and drinking patients in terms of the prognostic value related to HPV infection in HNSCCs. In fact, many studies suggest that HPV-positive HNSCC patients have better overall survival than do HPV-negative patients, suggesting that HPV-infected patients could have better prognoses [7, 8]. On the other hand, our research lab showed that HPV-positive HNSCC patients have a lower response to concomitant chemoradiotherapy and a decreased 5-year disease-free survival rate than do HPV-negative patients, highlighting their poorer prognoses [9, 10]. In fact, it appears that the biology of HNSCCs is more complex than we know, underlying that we must consider both HPV status (transcriptionally active or not) and classical risk factors (i.e., tobacco and alcohol consumption) [11].

The host immune system plays a critical role in the development and progression of HNSCCs. Among immune cells, macrophages constitute strong mediators of inflammatory responses, particularly in the fight against cancer [12, 13]. Depending on the tumor environment stimuli, macrophages present two different phenotypes. Macrophages of the M1 phenotype contribute to cytotoxic CD8+ T cell activation and naïve CD4+ T cell differentiation into Th1 effector cells, leading to antitumor effects [14–16]. Among M2 macrophages, tumor-associated macrophages (TAMs) stimulate regulatory T cell differentiation and secrete several factors (e.g., TGF-β, TNF-α and IL-10) to create a favorable environment for tumor growth and immunosuppression promotion [17, 18].

In squamous cell carcinomas, recent studies found a positive correlation between CD68+ macrophages (both M1 and M2 phenotypes) and tumor progression in cervical cancers [19–21]. Moreover, a high number of M1 macrophages appears to be an independent prognostic factor for longer survival in patients with cervical carcinoma [22]. Finally, several studies have described a positive correlation between M2 macrophage infiltration and tumor progression of HNSCCs [23].

In the same way, HPV infection plays an important role in tumor progression by modulating the tumor immune environment in order to promote tumor escape. In fact, our previous studies showed that Langerhans cell infiltration is a significant prognostic factor for HNSCCs and that the number of these immune cells is decreased in HPV-positive HNSCCs [24]. Likewise, we demonstrated increased regulatory T-cell numbers in HPV-related HNSCCs [25, 26]. Moreover, HPV interacts with CD68+ macrophages by recruiting them to the tumor site. Lepique *et al.* observed a high CD68+ macrophage infiltration rate in an animal model of HPV16-E6/E7-induced tumors, and this macrophage population was mainly constituted of TAMs [27]. Finally, the density of CD68+ macrophages appears to be higher in the tumor area (but not in the stromal regions) of HPV-positive oropharyngeal squamous cell carcinomas (OPSCCs) than in HPV-negative OPSCCs [28].

Here, we evaluated the CD68+ macrophage numbers during HNSCC progression in a large clinical series composed of 10 cases of tumor-free peri-tumoral epithelia (TFE), 43 cases of low-grade dysplasia (LGD), 45 cases of high-grade dysplasia (HGD) and 110 cases of carcinoma (CA), including 74 cases of HPV-negative and 36 cases of HPV-positive HNSCCs. We also assessed the prognostic value of CD68+ macrophage numbers in the stromal and intra-epithelial compartments of these patients, compared to other classical risk factors such as tobacco and alcohol consumption, HPV status and tumor stage. Finally, we conducted *in vivo* studies to investigate whether HPV16-E6 and –E7 oncoproteins modulate CD68+ macrophage recruitment in an orthotopic mouse model of HNSCC.

## RESULTS

### CD68+ macrophage number is associated with poor prognosis in HNSCC

**Figure 1:**
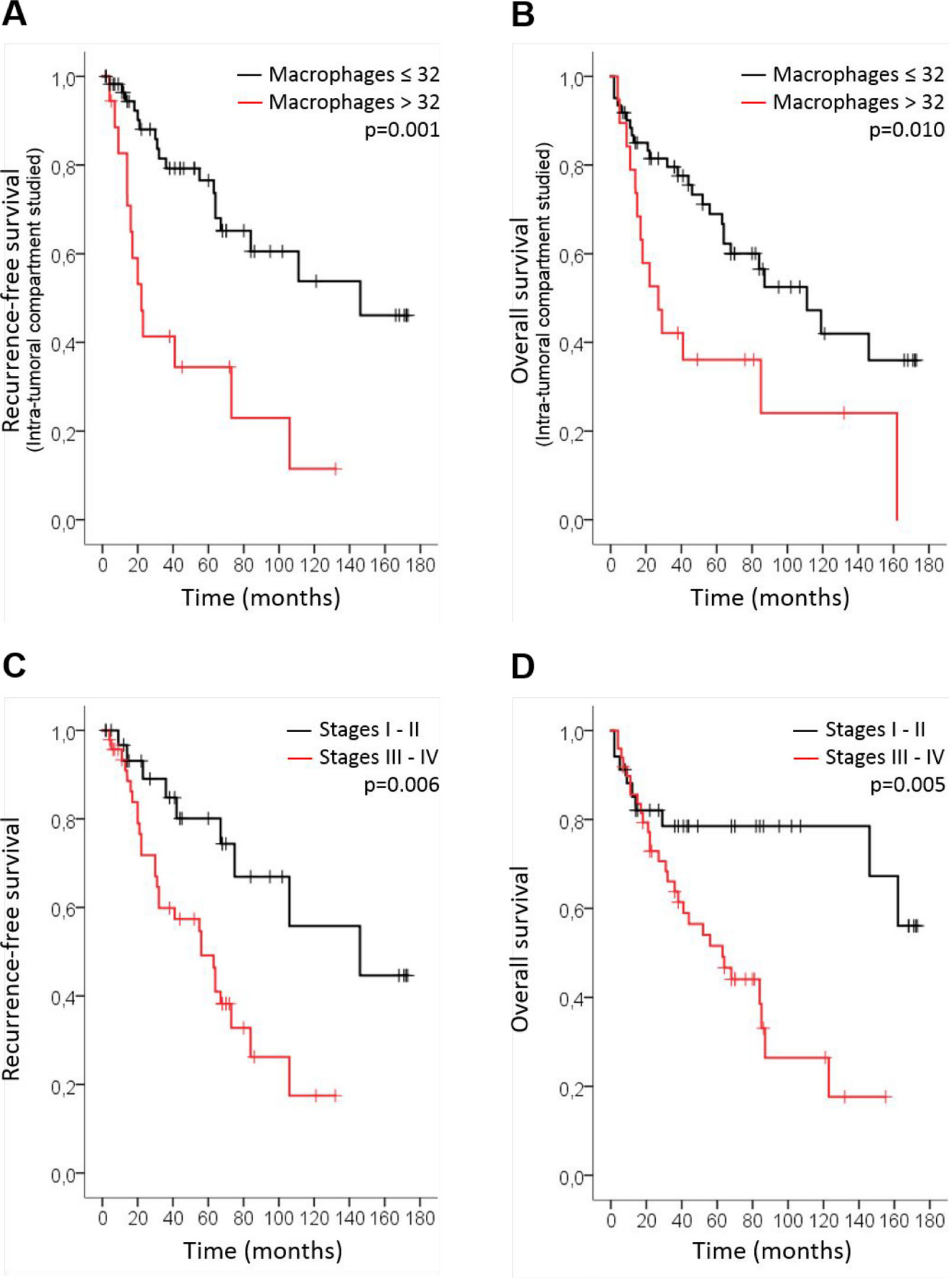
CD68+ macrophage number is associated with poor prognosis in HNSCC Kaplan-Meier curves of the recurrence-free survival (RFS) (cutoff at 32) (log-rank test, *p* = 0.001) (**A**) and overall survival (OS) (cutoff at 32) (log-rank test, *p* = 0.01) (**B**) of patients with HNSCC in the intra-tumoral compartment according to the number of CD68+ macrophages. Kaplan-Meier curves of the RFS (log-rank test, *p* = 0.006) (**C**) and OS (log-rank test, *p* = 0.005) (**D**) according to tumor stage.

We assessed the overall survival (OS) rate and the recurrence-free survival (RFS) rate of patients with HNSCC in intra-tumoral and stromal compartments according to CD68+ macrophage numbers. We first used the Cutoff finder web application to estimate the optimal cutoff point for the CD68+ macrophage population; we found that 32 (when evaluating the intra-tumoral compartment) and 67 (when evaluating the stromal compartment) were the optimal cutoff values for CD68+ macrophage numbers. In the intra-tumoral compartment, a high number of CD68+ cells (>32) was statistically associated with a poorer prognosis in terms of RFS (log-rank test, *p* = 0.001) (Figure 1A) and OS of patients with HNSCC (log-rank test, *p* = 0.01) (Figure 1B). However, no significant correlation between CD68+ macrophage number and prognosis was found regarding OS and RFS in the stromal compartment.

Regarding tumor stage, patients with advanced stages (III-IV) had shorter RFS (*p* = 0.006) (Figure 1C) and OS (*p* = 0.005) than did other patients (Figure 1D). Finally, multivariate analyses indicated that CD68+ macrophage number (*p* = 0.002 RFS; *p* = 0.016 OS) and tumor stage (*p* = 0.014 RFS; *p* = 0.014 OS) had more significant prognostic value than did classical risk factors as such as tobacco, alcohol and HPV status (Table 1).

**Table 1:** Univariate and multivariate Cox regression models evaluating the involvement of CD68+ macrophage number, tobacco, alcohol, tumor stage and HPV status on recurrence-free survival (RFS) and overall survival (OS)

	RFS		OS	
Variables	HR (95% CI)	*p* value	HR (95% CI)	*p* value
**Univariate analysis**				
CD68+ macrophage number (<32 vs >32)	3.638 (1.755–7.539)	**0.001**	2.353 (1.222–4.531)	**0.010**
Tobacco (non smoker vs smoker)	1.570 (0.691–3.568)	0.281	1.647 (0.728–3.726)	0.231
Alcohol (non drinker vs drinker)	1.534 (0.763–3.084)	0.230	1.392 (0.692–2.801)	0.353
Tumor stage (I–II vs III–IV)	3.017 (1.363–6.677)	**0.006**	3.166 (1.414–7.089)	**0.005**
HPV status (HPV– vs HPV+)	0.517 (0.184–1.457)	0.212	1.020 (0.472–2.205)	0.959
**Multivariate analysis**				
CD68+ macrophage number (<32 vs >32)	4.210 (1.672–10.602)	**0.002**	2.833 (1.218–6.589)	**0.016**
Tobacco (non smoker vs smoker)	0.885 (0.201–3.899)	0.872	1.272 (0.348–4.646)	0.716
Alcohol (non drinker vs drinker)	1.159 (0.360–3.734)	0.805	0.640 (0.238–1.721)	0.377
Tumor stage (I–II vs III–IV)	3.622 (1.293–10.147)	**0.014**	3.519 (1.288–9.616)	**0.014**
HPV status (HPV– vs HPV+)	0.306 (0.085–1.100)	0.070	0.378 (0.110–1.303)	0.123

### CD68+ macrophages are correlated with tumor stage, tumor location and HPV status

**Figure 2:**
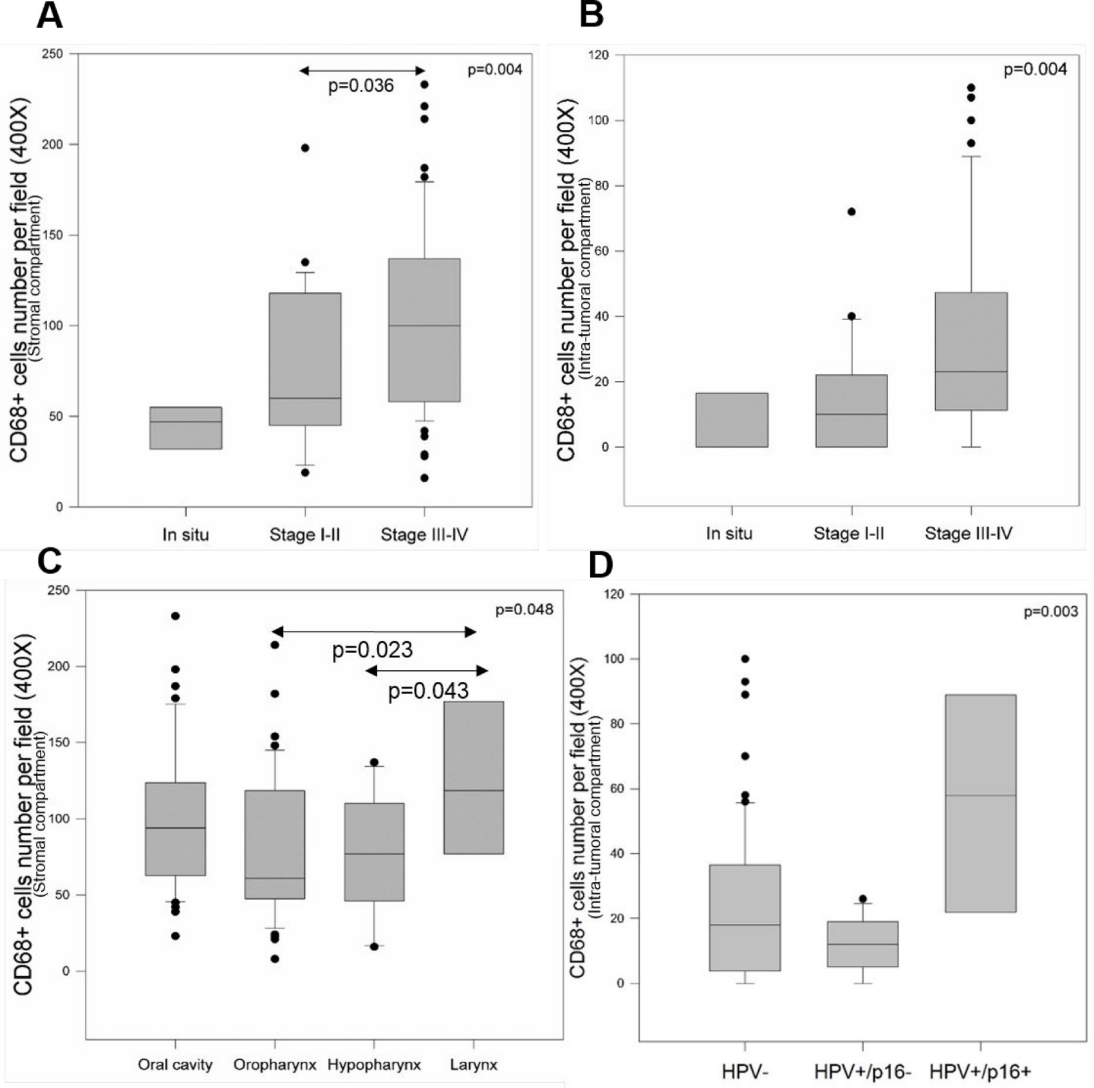
CD68+ macrophages are correlated with tumor stage, tumor location and HPV status Evaluation of CD68+ macrophage number in stromal regions (**A**) and intra-tumoral regions (**B**) of *in situ* lesions, stages I-II tumors and stages III-IV tumors (Kruskal–Wallis test, *p* = 0.004). Evaluation of CD68+ macrophage number in the stromal compartment of tumors of the oral cavity, oropharynx, hypopharynx and larynx (Kruskal–Wallis test, *p* = 0.048) (**C**). Evaluation of CD68+ macrophage number in the intra-tumoral compartment of HPV, HPV+/p16− and HPV+/p16+ tumors (Kruskal–Wallis test, *p* = 0.003) (**D**).

The association between CD68+ macrophage number and age, gender, tumor location, histological grade, tumor stage, tobacco and alcohol consumption and HPV status was evaluated in both the intra-tumoral and stromal compartments of 110 HNSCC samples (Table 2). No significant correlation was observed regarding CD68+ macrophage number and age, gender, histological grade, tobacco and alcohol consumption in either compartment. However, we noticed a significant correlation between CD68+ macrophages and tumor stage (*p* = 0.004), showing a higher number of these cells in the stromal compartment of advanced-stage tumors (III and IV) (Figure 2A), such as in intra-tumoral regions (Figure 2B). Moreover, when evaluating the stromal compartment, CD68+ macrophage density was significantly different depending on the observed tumor location (Kruskal–Wallis test, *p* < 0.05). CD68+ macrophage numbers were significantly higher in larynx than in hypopharynx (*p* = 0.043) and oropharynx carcinomas (*p* = 0.023) (Figure 2C). No differences in CD68+ macrophage numbers were observed between the several locations studied when looking at the intra-tumoral compartment. Concerning HPV status, CD68+ macrophage numbers were statistically higher in the intra-tumoral compartment of HPV+/p16+ tumors than in HPV+/p16− and HPV- tumors (*p* = 0.003) (Figure 2D).

**Table 2:** Characteristics of patient population used in this study

Variables	Number of cases (*N* = 110)	*p* value CD68 in intra-tumoral compartment	*p* value CD68 in stromal compartment
**Age (years)**		0.489	0.461
Median	58		
Range	37–88		
**Gender**		0.122	0.359
Male	81		
Female	29		
**Anatomic site**		0.297	**0.048**
Oral cavity	42		
Oropharynx	48		
Larynx	8		
Hypopharynx	11		
Nasopharynx	1		
**Histological grade (differentiation)**		0.920	0.887
Well	52		
Moderate	43		
Poor	3		
Unknown	12		
**Tumor stage**		**0.004**	**0.004**
* In situ*	2		
I–II	37		
III–IV	56		
Unknown	15		
**Risk factors**			
*** Alcohol***		0.652	0.120
Drinker	45		
Non drinker	33		
Unknown	32		
*** Tobacco***		0.213	0.675
Smokers	58		
Non smokers	30		
Unknown	22		
*** HPV status***		0.445	0.197
Negative	82		
Positive (p16–)	12		
Positive (p16+)	6		
Positive (p16 unknown)	10		
**Recurrence-free survival (RFS)**		**0.001**	0.679
Yes	32		
None	52		
Unknown	26		
**Overall survival (OS)**		**0.010**	0.816
Dead	43		
Alive	48		
Unknown	19		

### CD68+ macrophage infiltration increases during HNSCC progression

**Figure 3:**
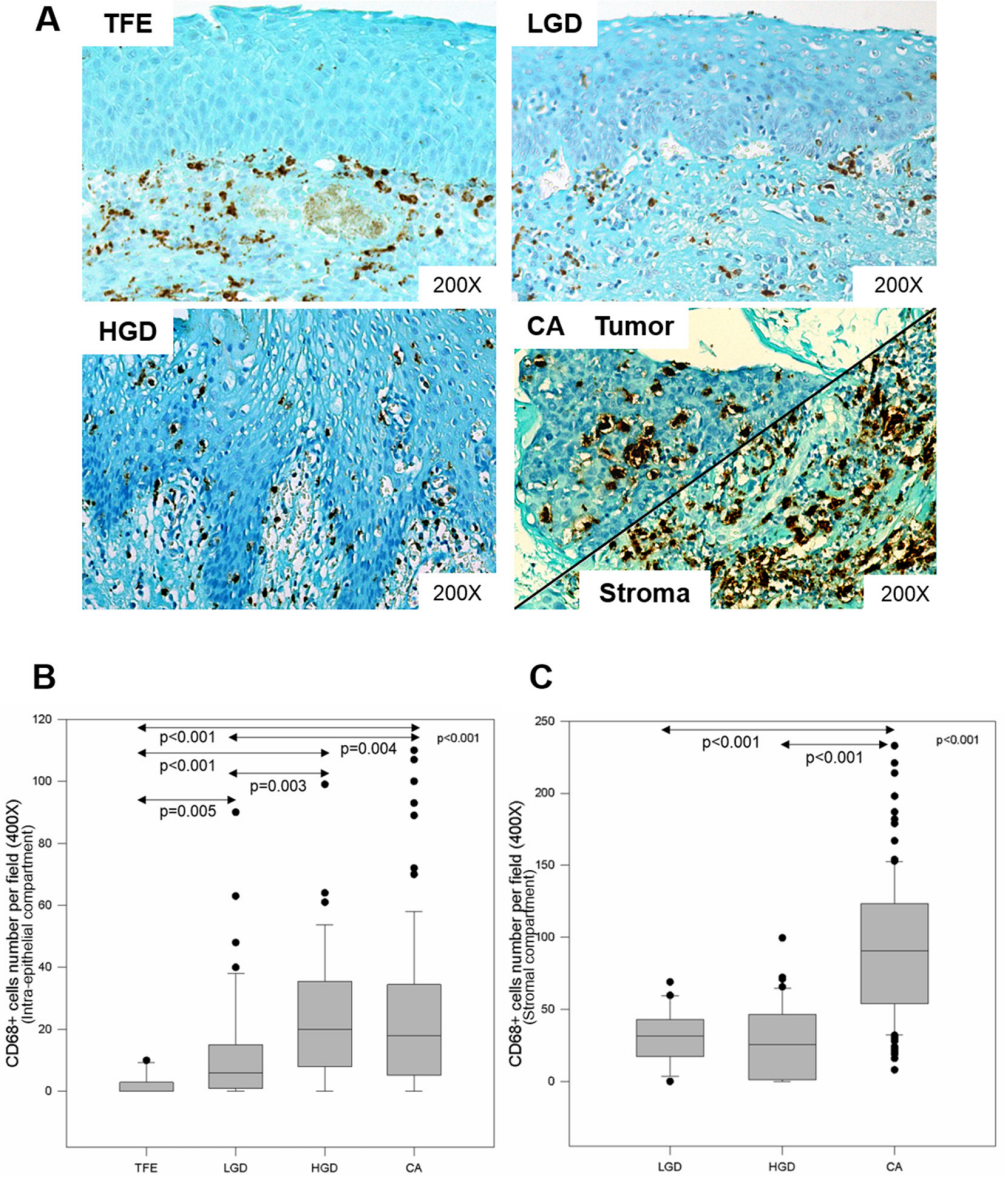
CD68+ macrophage infiltration increases during HNSCC progression Immunohistochemical representation of CD68 during HNSCC progression in epithelial and stromal compartments, from tumor-free peri-tumoral region, low-grade dysplasia, high-grade dysplasia to carcinoma region (**A**). CD68+ macrophage number during tumor progression in the intra-tumoral compartment (Kruskal–Wallis test, *p* < 0.001) (**B**) and the stromal compartment (Kruskal–Wallis test, *p* < 0.001) (**C**).

**Figure 4:**
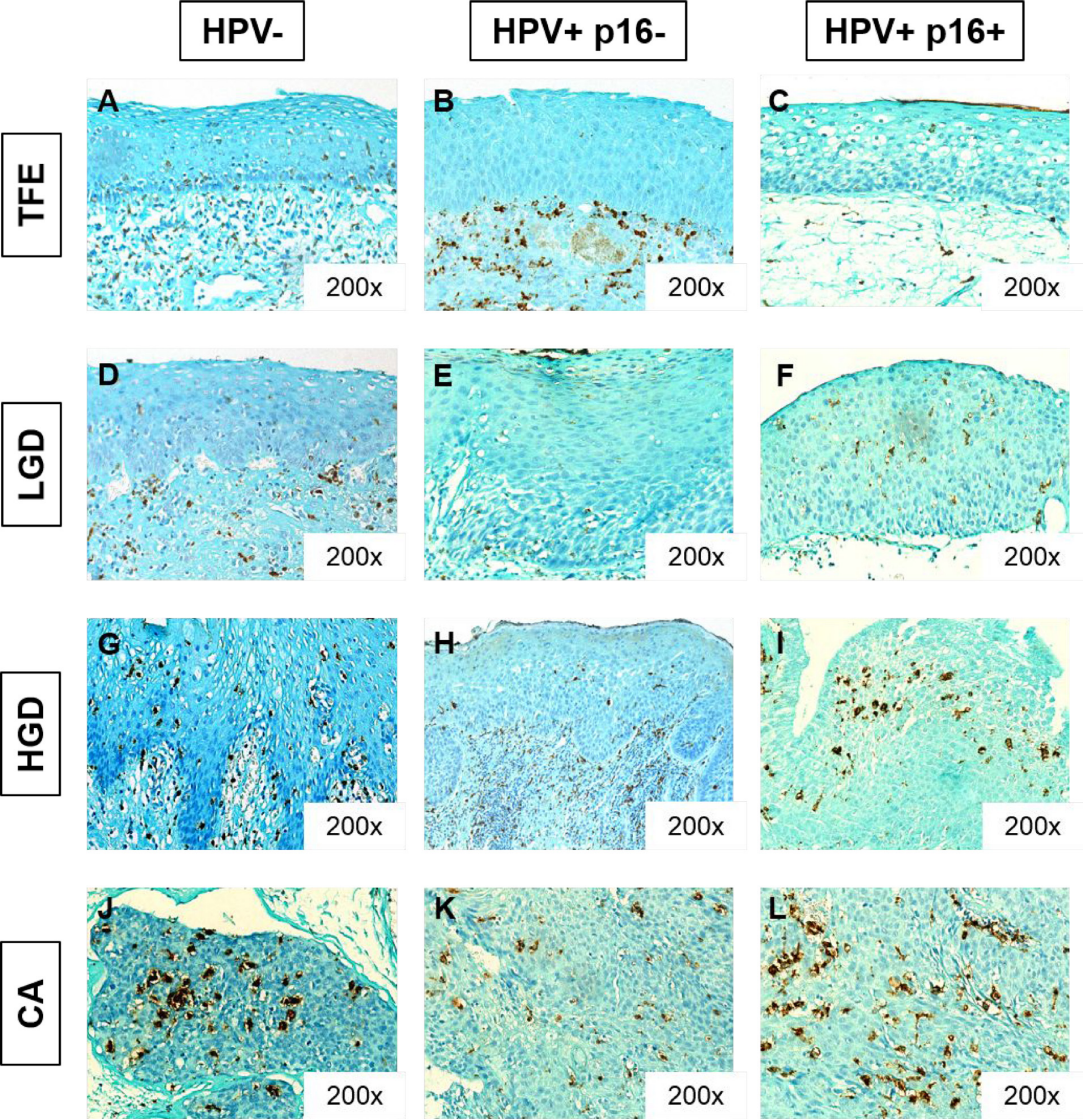
CD68+ macrophage infiltration increases during HNSCC progression, despite HPV status Immunohistochemical representation of CD68 in tumor-free peritumoral epithelium (TFE) (**A**, **B**, **C**), low-grade dysplasia (LGD) (**D**, **E**, **F**), high-grade dysplasia (HGD), (**G**, **H**, **I**) and carcinoma (CA) (**J**,**K**,**L**) from HPV- patients (A, D, G, J), HPV+/p16- patients (B, E, H, K) and HPV+/p16+ patients (C, F, I, L).

**Figure 5:**
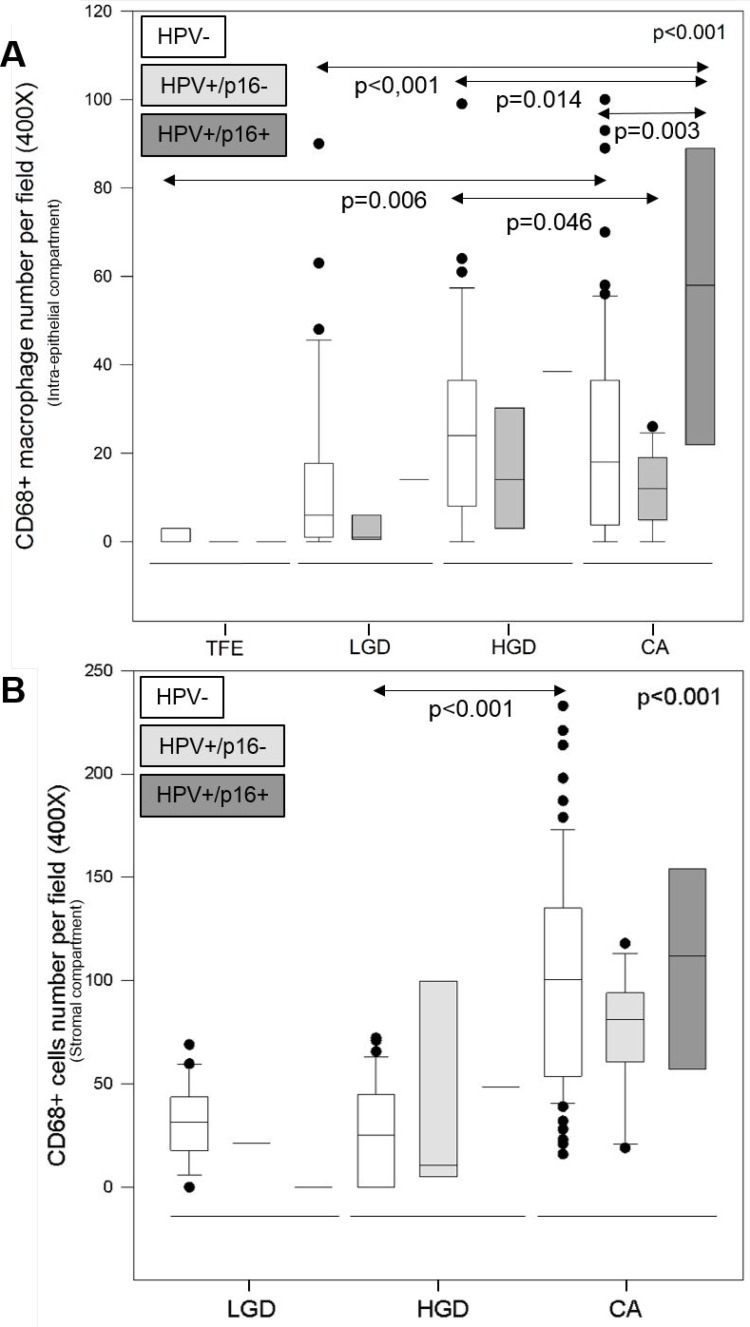
CD68+ macrophage infiltration is higher in HPV+/p16+ HNSCC CD68+ macrophage number during tumor progression according to HPV status in the intra-tumoral compartment (Kruskal–Wallis test, *p* = 0.003) (**A**) and the stromal compartment (Kruskal–Wallis test, *p* < 0.001) (**B**).

We evaluated CD68+ macrophage numbers in 10 cases of tumor-free peri-tumoral epithelia (TFE), 43 cases of low-grade dysplasia (LGD), 45 cases of high-grade dysplasia (HGD) and 110 cases of carcinoma (CA). We observed increased CD68+ macrophage recruitment during disease progression (from TFE to CA) in both the intra-epithelial compartment and in the stromal compartment (Figure 3A). Kruskal–Wallis test revealed that this increase was statistically significant in both compartments (*p* < 0.001) (Figures 3B–3C). In addition, we noticed a higher number of CD68+ macrophages in stromal regions (max. 240 cells per field, 400X magnification) than in epithelial regions (max. 110 cells per field) (Figures 3B–3C). In the intra-epithelial compartment, CD68+ macrophage numbers were significantly different between TFE and LGD (post hoc test, *p* = 0.005), TFE and HGD (*p* < 0.001), and TFE and CA (*p* < 0.001). Moreover, there was a statistically significant difference between LGD and HGD (*p* = 0.003) and LGD and CA (*p* = 0.004) (Figure 3B). In the stromal compartment, we noticed a significant difference between dysplasia (low-grade and high-grade) and carcinoma (post hoc test, *p* < 0.001) (Figure 3C). Concerning HPV status, CD68+ macrophage numbers increased during disease progression, despite HPV status (Figure 4) but was significantly higher in the intra-tumoral compartment of HPV+/p16+ tumors than in HPV+/p16− and HPV- tumors (*p* = 0.003) (Figure 5A). This difference was not statistically significant in the stromal compartment (Figure 5B).

### CD68+ macrophage recruitment is decreased by HPV16-E6 oncoprotein

To determine the implication of HPV16-E6 and –E7 oncoproteins in CD68+ macrophage recruitment, we inoculated SCC-VII cells transfected with HPV16-E6, −E7, −E6/E7 or not (control, CT) in female C3H/HeN mice. First, CD68+ macrophage numbers were evaluated in tumor samples derived from the four groups of mice and appeared to be significantly different (Kruskal–Wallis test, *p* = 0.006). Indeed, we observed a higher number of CD68+ macrophages in the control group compared to the E6 group (*p* = 0.012), and a significantly higher CD68+ macrophage number in the E7 and E6/E7 groups compared to E6 alone (*p* = 0.029 and *p* < 0.001) (Supplementary Figure 1). This observation is in accordance with the invasion assay performed by co-culturing the RAW macrophage cell line with SCC-VII-CT, −E6, −E7 and –E6/E7. Indeed, the number of invaded macrophages was higher when exposed to SCC-VII-CT than to –E6, and higher in –E7 and –E6/E7 compared to –E6 alone; however, this observation was not statistically significant (*p* = 0.085) (Supplementary Figure 2).

### CD206 immunostaining in SCC-VII E6/E7 mouse tumors

After staining the entire macrophage population in SCC-VII tumors, we wanted to distinguish the proportion of M1 and M2 macrophages in these tumors. We used CD206 immunostaining to detect M2 (TAMs) macrophages. We observed good CD206 staining in mouse lung tissue (positive control tissue), but no CD206+ macrophages were detected in SCC-VII CT, −E6, −E7 or –E6/E7 tumors (Supplementary Figure 3).

## DISCUSSION

Macrophages represent critical mediators of inflammatory processes induced by innate and adaptive immune systems. They also play an important role in HNSCC progression, whether by promoting it (for M2 tumor-associated macrophages) or by repressing it (for M1 macrophages). The main marker used in immunohistochemistry to detect both M1 and M2 macrophages is CD68 [30, 31]. In this study, we showed that CD68+ macrophage infiltration significantly increases during head and neck tumor progression, both in the intra-epithelial compartment and in the stromal compartment. Indeed, we observed a significantly higher density of CD68+ macrophages in carcinoma compared to dysplasia (high-grade and low-grade) and to tumor-free peri-tumoral epithelia. If we consider the stromal compartment, we observed a higher number of CD68+ macrophages in the stromal region of carcinoma than in the stromal region under dysplasia, and these differences were statistically significant. These results are in accordance with several studies demonstrating significantly increasing CD68+ macrophage infiltration from oral normal mucosa to oral squamous cell carcinoma (OSCC), suggesting that CD68 immunostaining could be an important diagnostic and prognostic factor for OSCCs [30, 32, 33].

Our study also showed correlations between CD68+ macrophage infiltration and some clinical data. Indeed, we found a significant correlation between CD68+ macrophage number and tumor stage, with the density of CD68+ macrophages being higher in advanced stages (III and IV) in both intra-tumoral and stromal compartments. This was also observed in oropharyngeal squamous cell carcinomas and cervical carcinomas, where CD68+ macrophage infiltration was independently associated with tumor stage [19, 34]. Moreover, we showed a significant correlation between CD68+ macrophage numbers and tumor location. The density of CD68+ macrophages was significantly higher in the stroma of laryngeal tumors than in the stromal compartment of hypopharyngeal and oropharyngeal carcinomas. This result is in accordance with the study of Hu *et al.*, which described a difference in CD68+ macrophage infiltration in different locations of oral squamous cell carcinomas [35].

When evaluating the recurrence-free survival rate and the overall survival rate of patients with HNSCCs according to CD68+ macrophage infiltration, our results showed that a high density of CD68+ macrophages inside the tumor was associated with shorter recurrence-free and overall survival of HNSCC patients. These results are in accordance with other studies demonstrating that high CD68+ macrophage infiltration was associated with shorter overall survival in head and neck squamous cell carcinomas [35–37]. Likewise, multivariate analyses showed that CD68+ macrophage infiltration, as well as tumor stage, had more significant prognostic values than did other classical risk factors, supporting that CD68+ macrophage density in the intra-tumoral compartment of HNSCCs is a strong and independent prognostic factor for HNSCC patients. These observations could be explained by hypothesizing that the macrophage population in HNSCCs is mainly composed of M2 tumor-associated macrophages.

Regarding HPV status, we noticed in the intra-tumoral compartment that CD68+ macrophage infiltration was higher in transcriptionally active HPV+ HNSCCs than in HPV+/p16- and HPV- HNSCCs. This is consistent with Oguejiofor *et al.*, whose study used immunohistochemistry to demonstrate significantly higher CD68+ macrophage recruitment in the tumor area of HPV+ oropharyngeal squamous cell carcinomas than in HPV- carcinomas [28].

To better understand the involvement of HPV oncoproteins in CD68+ macrophage recruitment to the tumor, we developed an orthotopic female mouse model injected with squamous cell carcinoma cell lines transfected with HPV16-E6, −E7 or –E6/E7. Our *in vivo* study showed significantly higher CD68+ macrophage infiltration in SCC-VII-E7+ (median = 0.2) and SCC-VII-E6/E7+ (median = 0.4) than in SCC-VII-E6+ tumors (median = 0). The same result was obtained when co-culturing macrophages with SCC-VII-E6/E7+ during the invasion assay. Indeed, the number of invaded macrophages appeared to be higher when exposed to –E7 and –E6/E7 oncoproteins than to –E6 alone. However, CD68+ macrophage infiltration and invasion are the highest in the SCC-VII CT mouse tumors and cell line, which would suggest that HPV-negative mouse tumors have higher CD68+ macrophage recruitment than do tumors transfected with HPV-E6 and –E7 oncoproteins (considered HPV-positive tumors). This observation in mouse tumors does not seem to be in accordance with the results obtained in human tumors, where CD68+ macrophage density was the highest in HPV+ HNSCCs. We could try to explain these conflicting results by comparing the two biological models. First, CD68+ macrophage numbers are much higher in human tumors (max 110) than in mouse tumors (max 3,5), highlighting the deeper interaction between stroma and tumor cells in the human model. In fact, mice were injected with a cancer cell suspension that led to the local establishment of a tumor mass, most often composed of agglomerated and necrotic tumor cells that did not invade the neighbor microenvironment. Second, in the human tumor model, the tumors developed spontaneously, tumor cells invaded the local micro-environment and tumor samples were composed of tumor cells interlaced with stroma and blood vessels. Thus, comparing these two biological models is complex because we cannot distinguish the intra-epithelial region from the stromal region in the mouse model. Third, SCC-VII cells injected in the mouse model were only transfected with HPV16-E6 and –E7 oncoproteins, while human HNSCC tumor cells are infected with the full HPV virus, which is composed of six early genes and two late genes [38]. CD68+ macrophage recruitment could be led by another HPV protein than E6 and E7. Indeed, each protein of the virus plays a role in immune system modulation, as detailed by Sasagawa *et al.* in cervical cancer [39]. Finally, the significantly higher CD68+ macrophage density in SCCVII-E7+ cells compared to SCC-VII-E6+ cells highlights the ability of E7 to interact differently with the immune system than E6. Indeed, our previous study in the same *in vivo* model of HNSCC demonstrated that FoxP3 T cell infiltration was significantly higher in SCC-VII-E7+ tumors, suggesting that HPV16-E7 oncoprotein might be a key factor in the regulatory T cell recruitment in mice [26].

In conclusion, we demonstrate for the first time that CD68+ macrophage infiltration increases during head and neck tumor progression, both in the intra-epithelial and in the stromal compartment. Moreover, high CD68+ macrophage density and advanced tumor stage are associated with shorter recurrence-free and overall survival of HNSCC patients. Our study suggested that CD68+ macrophage infiltration is higher in transcriptionally active HPV+ HNSCCs than in HPV- HNSCCs.

## MATERIALS AND METHODS

### Population and clinical data

Formalin-fixed, paraffin-embedded HNSCC specimens were obtained from 110 patients who underwent curative surgery at CHU Sart-Tilman (Liège, Belgium) and EpiCURA Baudour Hospital (Baudour, Belgium) during the years 2001 to 2011. Among these patients, 82 (74%) were not infected with HPV, 12 (11%) were infected with a transcriptionally non-active HPV (HPV+/p16-) and 6 (5%) were infected with a transcriptionally active HPV (HPV+/p16+). Based on tobacco and/or alcohol consumption, patients were classified as smokers and non-smokers as well as drinkers and non-drinkers at the time of the HNSCC diagnosis (Table 1). This retrospective study was approved by the Institutional Review Board.

### DNA extraction

Formalin-fixed, paraffin-embedded tissue samples were sectioned (10 × 5 µm), deparaffinized and digested with proteinase K overnight at 56° C. DNA was extracted using the QIAamp DNA Mini Kit (Qiagen, Benelux, Belgium) as previously described [10].

### Detection of HPV by polymerase chain reaction (PCR) amplification

HPV DNA detection was performed using PCR with GP5+/GP6+ primers (synthesized by Eurogentec, Liege, Belgium) that amplify a consensus region located within the L1 region of the HPV genome, as previously described [10].

### Immunohistochemistry

#### p16 immunostaining

All HPV+ samples were immunohistochemically evaluated for p16 expression using the recommended mouse monoclonal antibody (CINtec p16 (clone E6H4), Ventana, Tucson, AZ, USA) and an automated immunostainer (Bond-Max, Leica Microsystems, Wetzlar, Germany), as previously described [24]. The expression of p16 was defined as positive only when both nucleus and cytoplasm were stained and when more than 70% of tumor cells were stained.

#### CD68 immunostaining for human tissue

CD68+ macrophages were detected in human tissues by immunohistochemistry using a CD68 (PG-M1) mouse monoclonal antibody (Dako, Glostrup, Denmark) at a dilution of 1:200. First, the tumor samples were deparaffinized in two xylene baths and then rehydrated in four ethanol baths (decreasing concentrations from 100% to 70%). Next, the samples were immersed in a 4.5% H_2_O_2_/methanol bath and finally in distilled water. Epitope retrieval was performed by immersing the samples in EDTA buffer (Klinipath BVBA, Olen, Belgium), followed by heating in a pressure cooker. After epitope retrieval, primary antibody was incubated for one hour at room temperature. Finally, the samples were incubated with a PowerVision Poly-HRP-anti-mouse IgG (Klinipath, Duiven, Holland), and the antigens were visualized via the addition of a solution of 3–3′ DAB- H_2_O_2_-EDTA buffer (Liquid DAB, San Ramon, USA) before coloring with Mayer’s hemalum (Klinipath, Duiven, Holland) and mounting with a synthetic balm (Thermo Scientific, Pittsburg, PA, USA). To exclude antigen-independent staining, controls for which the incubation step with the primary antibody was omitted were examined. In all cases, these controls were negative. The number of CD68+ macrophages was counted in 5 fields in each area (TFE, LGD, HGD, CA) with an AxioCam MRC5 optical microscope (Zeiss, Hallbergmoos, Germany) at 400X magnification.

#### CD68 immunostaining for mouse tissue

CD68+ macrophages were detected in mouse tissues by immunohistochemistry using a CD68 (FA-11) rat monoclonal antibody (ThermoFisher Scientific, Rockford, IL, USA) at a dilution of 1:200. After deparaffinization, epitope retrieval was performed by immersing the sections in citrate buffer (Scytek, UT, USA), followed by heating in a pressure cooker. Thereafter, sections were successively exposed to solutions containing avidin and biotin to avoid false-positive staining reactions resulting from endogenous biotin, followed by casein and anti-CD68 incubation overnight. The day after, sections were exposed to the corresponding biotinylated secondary antibody (polyclonal rabbit anti-rat IgG) and then to the avidin-biotin-peroxidase complex (ABC kit), both from Vectorlab (Peterborough, UK). Presence of antigen in the sections was visualized by incubation with a chromogenic substrate mixture containing DAB and H_2_O_2_. Finally, sections were counterstained with luxol fast blue and mounted with a synthetic medium. The number of CD68+ macrophages was counted in 5 fields in each tumor tissue with an AxioCam MRC5 optical microscope at 400× magnification.

### CD206 immunostaining for mouse tissue

CD206+ macrophages were distinguished in mouse tissues by immunohistochemistry using a CD206 (anti-mannose receptor) rabbit polyclonal antibody (Abcam, Cambridge, United Kingdom) at a dilution of 1:250. The same steps as for CD68 immunostaining were done, but the immersion in H_2_O_2_/methanol was omitted, as recommended by the manufacturer’s protocol. Heat-mediated antigen retrieval was performed with citrate buffer, followed by an anti-CD206 incubation at room temperature. Finally, sections were exposed to the corresponding biotinylated secondary antibody (polyclonal donkey anti-rabbit IgG). The last steps of the procedure are similar to those of the CD68 immunostaining.

### Cell lines and culture conditions

Mouse SCCVII cells were transfected with 3 different vectors to express the HPV oncoproteins E6, E7 or both E6/E7 in the Radiation Oncology Department at the Université Catholique de Louvain (Prof. Vincent Grégoire), as previously described [26]. The RAW cell line is a murine macrophage cell line that originates from murine blood and is a kind gift from Prof. Alexandre Legrand (University of Mons). This cell line was cultured in DMEM high-glucose medium supplemented with 10% fetal calf serum (VWR International, Leuven, Belgium), 1% penicillin-streptomycin and 1% non-essential amino acids (ThermoFisher Scientific).

### *In vivo* studies

Animal studies were conducted on 80 female C3H/HeN mice (Charles River Laboratory, L’Arbresle, France). The animals were maintained and handled in compliance with the guidelines issued by the Belgian Ministry of Trade and Agriculture. A suspension of SCCVII cells transfected for E6, E7 and E6/E7 expression or not (control, CT) was injected in the mylohyoid muscle following a procedure detailed in a previous publication [29]. Animals were monitored for tumor onset and were euthanized when they exhibited a tumor more than 15 mm diameter or a weight loss of more than 20%.

### Involvement of HPV16-E6/E7 oncoproteins in macrophage invasion

Co-culture of the RAW cell line and SCC-VII-E6, −E7, −E6/E7 and –CT cells was done in quadruplicate by using Boyden chambers. SCC-VII cell lines were seeded in 12-wells plate (4 × 10^4^ cells per well) and cultured for 72 hours. After that, SCC-VII medium was renewed, and RAW cells were seeded in Boyden chambers (1 × 10^5^ cells per chamber) that were placed in the wells culturing SCC-VII cells for 24 hours. The last step consisted of staining the invaded macrophages with crystal violet.

### Statistical analyses

The medians of the independent data groups were compared using nonparametric Mann–Whitney test (2 groups) or Kruskal–Wallis test (>2 groups). When the latter test was significant, the Dunn post hoc test was used to compare each pair of groups (to avoid multiple comparison effects). The optimal cutoff points of the populations were estimated using Cutoff finder web application [40]. Recurrence-free survival and overall survival analyses were performed using Kaplan-Meier curves, and the results were compared with log-rank tests. Univariate and multivariate Cox regression models were applied to calculate the hazard ratio (HR) and 95% confidence interval (95% CI) and to assess the independent contributions of CD68+ macrophages to RFS and OS in the presence of other covariates, including tobacco, alcohol, HPV status and tumor stage. *P*-values < 0.05 were considered statistically significant. All statistical analyses were performed using SigmaPlot 11.0 (Systat Software, San Jose, CA, USA) and SPSS 23 (IBM, Chicago, IL, USA).

## SUPPLEMENTARY MATERIALS FIGURES


