# Supplementary material for: High infiltration of CD68+ macrophages is associated with poor prognoses of head and neck squamous cell carcinoma patients and is influenced by human papillomavirus
Source: Oncotarget. 2018 Jan 24;9(13):11046–59. doi: 10.18632/oncotarget.24306 (PMC5834277; doi:10.18632/oncotarget.24306)
Supplement: Supplementary file 1 [file oncotarget-09-11046-s001.pdf]

High infiltration of CD68+ macrophages is associated with poor prognoses of head and neck squamous cell carcinoma patients and is influenced by human papillomavirus

SUPPLEMENTARY MATERIALS

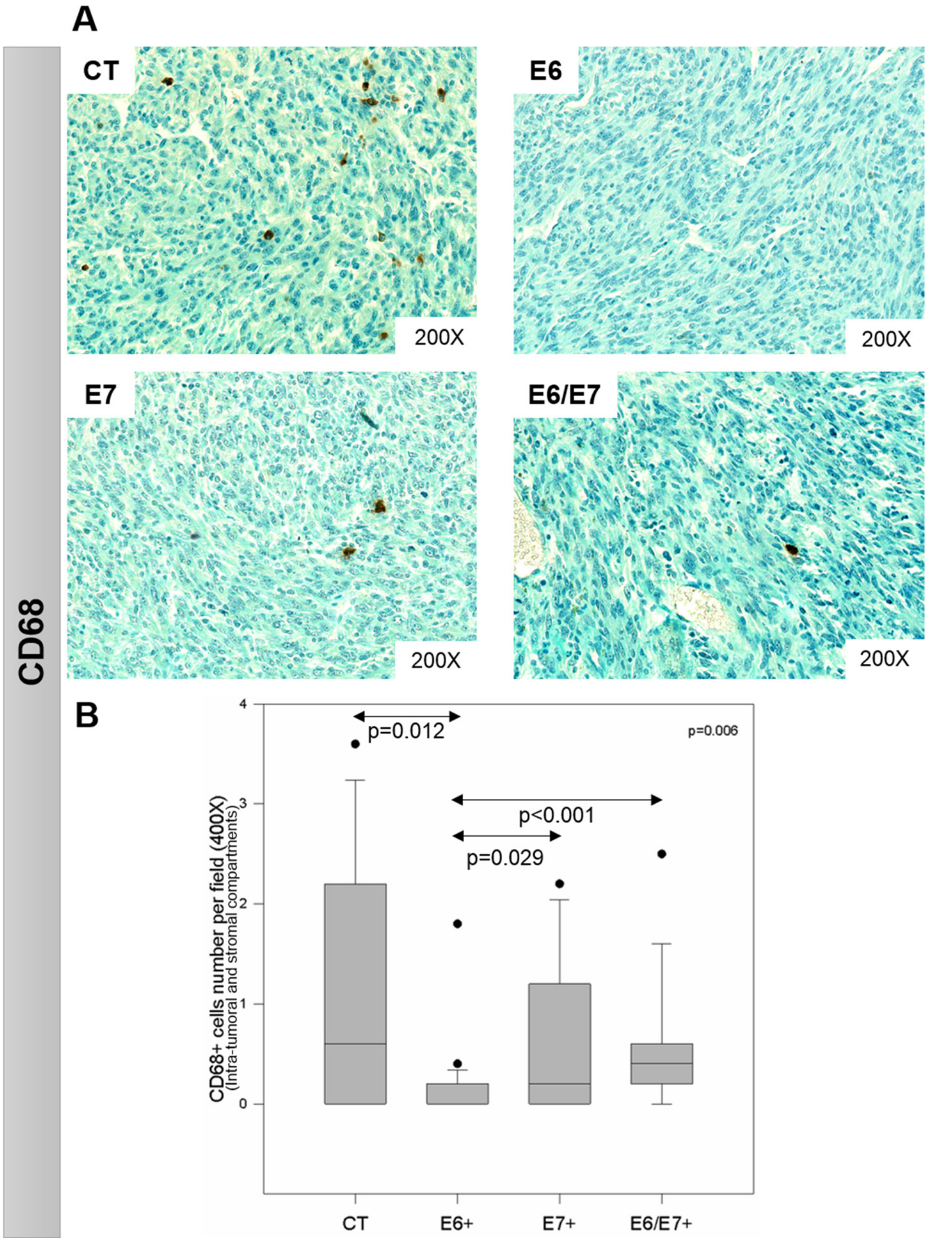

Supplementary Figure 1: CD68+ macrophage infiltration is decreased in SCC-VII E6 mouse tumors. Immunohistochemical representation of CD68 in control (CT), E6-, E7- and E6/E7-expressing tumors implanted in mice (**A**). Evaluation of CD68+ cells number in control (CT), E6-, E7- and E6/E7-expressing tumors (Kruskal-Wallis test, $p = 0.006$) (**B**).

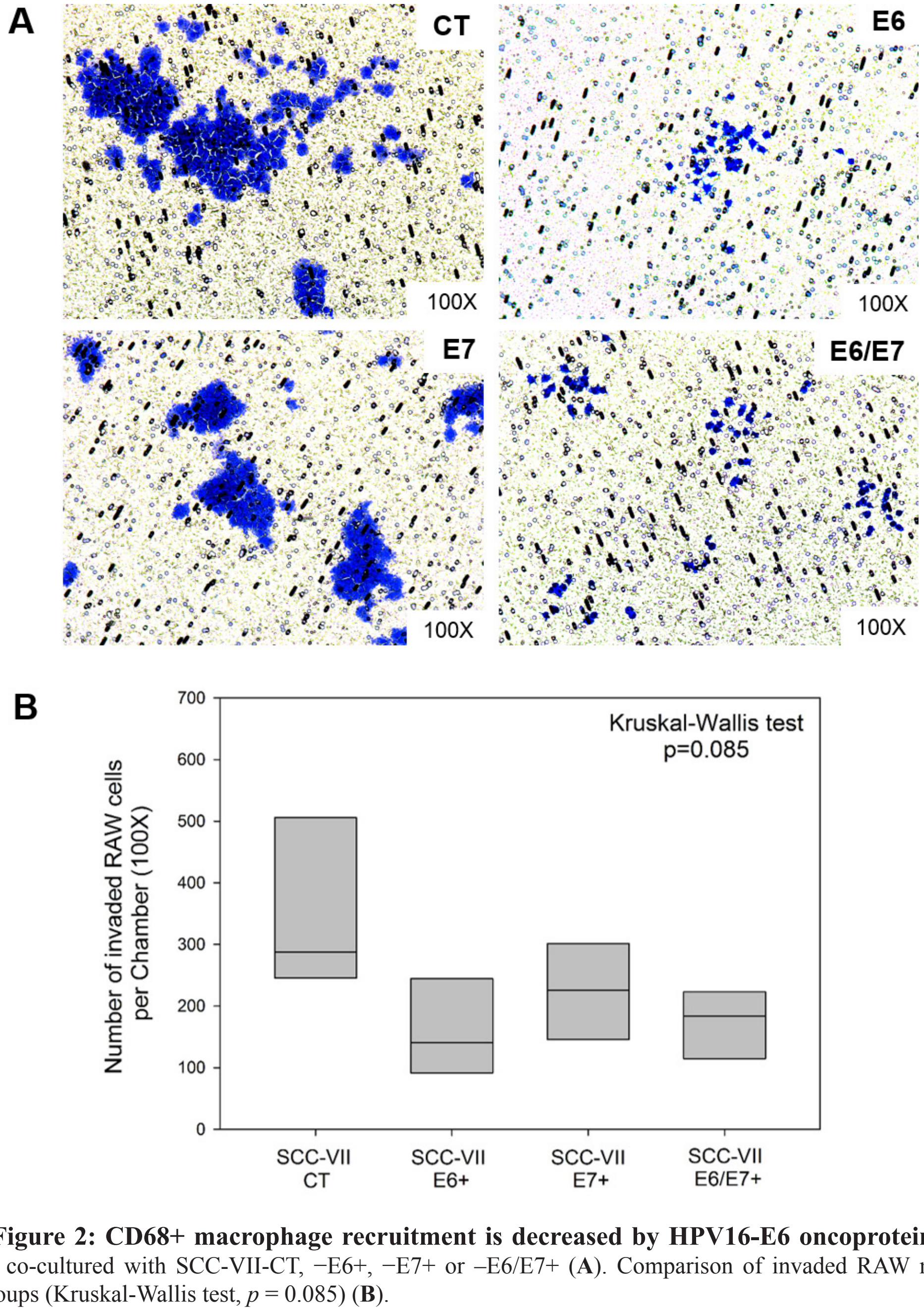

Supplementary Figure 2: CD68+ macrophage recruitment is decreased by HPV16-E6 oncoprotein. Invasion assay of RAW macrophages co-cultured with SCC-VII-CT, −E6+, −E7+ or –E6/E7+ (**A**). Comparison of invaded RAW macrophage number between the four groups (Kruskal-Wallis test, $p = 0.085$) (**B**).

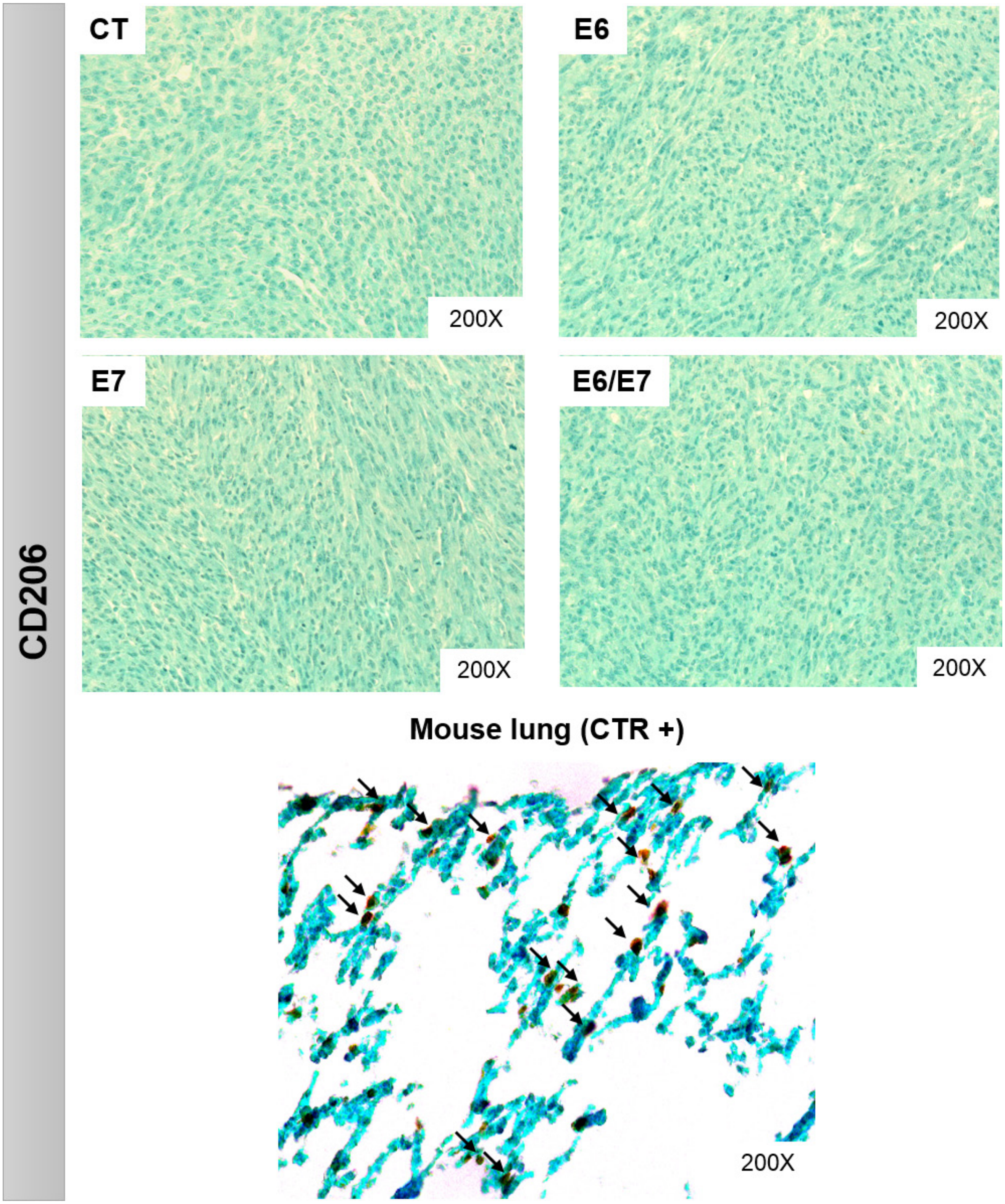

Supplementary Figure 3: CD206 immunostaining in SCC-VII E6/E7 mouse tumors. Immunohistochemical representation of CD206 in SCC-VII E6-, E7- and E6/E7-expressing tumors implanted in mice and in mouse lung control tissue (arrows).
